# THE INFLUENCE OF LONELINESS AND RURAL RESIDENCE ON DEPRESSION IN LATER LIFE

**DOI:** 10.1093/geroni/igz038.3051

**Published:** 2019-11-08

**Authors:** Na Sun, Cassandra Hua, Xiao Qiu, J Scott Brown

**Affiliations:** 1 Miami University, Oxford, Ohio, United States; 2 Miami University, Oxford, United States

## Abstract

Loneliness is associated with depression among older adults. Limited research has examined the role of rurality in relationship to loneliness and depression; the extant research has mixed findings. The socioemotional selectivity theory states that as people age the quality of relationships become more important than the quantity (English & Carstensen, 2016). Individuals in rural areas may have a low quantity of relationships but deeper social ties within the community; thus, they may be less likely to become depressed over time. The association between loneliness and depression may be amplified for people in non-rural areas because they are surrounded by other people but lack close relationships that are most important during the aging process. This study examines the effect of living in rural areas on loneliness on predicting baseline depression and loneliness, as well as changes in these outcomes over time. Data are from the 2006-2014 waves of Health Retirement Study. Regression models examine the relationship between depression loneliness and rural residence controlling for health conditions and demographic characteristics. Latent curve models examine the disparity in trajectories of loneliness and depressive symptoms by urban and rural residence. Older adults who feel lonely (p<.001) and in urban areas (p<.0.05) are more likely to be depressed. Furthermore, the effect of loneliness on depression is weakened by rural residence (p<.05). It is salient to understand the protective effect of rural residency on depression among older adults in the U.S. We discuss implications for policy.

